# Optimal Particle Filter Weight for Bayesian Direct Position Estimation in a GNSS Receiver

**DOI:** 10.3390/s18082736

**Published:** 2018-08-20

**Authors:** Jürgen Dampf, Kathrin Frankl, Thomas Pany

**Affiliations:** 1 Institute of Geodesy, Graz University of Technology, Steyrergasse 30, 8010 Graz, Austria; 2 Institute of Space Technology and Space Applications, Universität der Bundeswehr München, Werner-Heisenberg-Weg 39, 85577 Neubiberg, Germany; kathrin.frankl@unibw.de (K.F.); thomas.pany@unibw.de (T.P.)

**Keywords:** GNSS, Bayesian direct position estimation, BDPE, software receiver, particle filter, optimal particle weight

## Abstract

Direct Position Estimation (DPE) is a rather new Global Navigation Satellite System (GNSS) technique to estimate the user position, velocity and time (PVT) directly from correlation values of the received GNSS signal with receiver internal replica signals. If combined with Bayesian nonlinear filters—like particle filters—the method allows for coping with multi-modal probability distributions and avoids the linearization step to convert correlation values into pseudoranges. The measurement update equation (particle weight update) is derived from a standard GNSS signal model, but we show that it cannot be used directly in a receiver implementation. The numerical evaluation of the formulas needs to be carried out in a logarithmic scale including various normalizations. Furthermore, the residual user range errors (coming from orbit, satellite clock, multipath or ionospheric errors) need to be included from the very beginning in the stochastic signal model. With these modifications, sensible probability functions can be derived from the GNSS multi-correlator values. The occurrence of multipath yields a natural widening of the probability density function. The approach is demonstrated with simulated and real-world Binary Phase Shift Keying signals with 1.023 MHz code rate (BPSK(1)) within the context of a real-time software based Bayesian DPE receiver.

## 1. Introduction

The Position, Velocity and Time (PVT) estimates of a Global Navigation Satellite System (GNSS) receiver are sometimes not sufficiently accurate, in particular in difficult environments such as urban areas, forests or indoors. In such environments, the observations do not follow a Gaussian distribution. This fact adds a bias to the PVT solution, which can be generally described as close to Gaussian distributed [1,2,3,4,5]. Thus, the PVT estimation in difficult environments can benefit from Sequential Monte Carlo (SMC) methods because they consider all available statistical information in the estimation process.

Particle filters [6,7] belong to the group of non-parametric Bayesian filters and are an implementation of an SMC method [8]. Thus, they allow for nonlinear and non-Gaussian distributed system and observation models. The performance comparison between particle filters and the Extended Kalman Filter (EKF) within a nonlinear and non-Gaussian environment shows that improved position estimates can be expected using particle filters with the drawback of computational intensive processing [9].

The application of particle filters in the field of PVT estimation can mostly be found in research. Often, particle filters are applied in tracking multiple objects (see e.g., [10]). For this purpose, particle filters are used to combine different sensors with the GNSS receiver PVT estimate or the receiver observables, pseudorange and Doppler. For instance, the shadow matching algorithm is based on a particle filter that aims to improve the positioning results in dense urban areas by fusing three-dimensional geometry information of buildings with the PVT estimates from GNSS signals [11]. In [12], the indoor positioning results are improved by a particle filter that fuses PVT estimation from GNSS signals with high power 4G Long Term Evolution (LTE) mobile network signals compared to GNSS signals. Furthermore, in [13], a particle filter is used to improve the position estimate to support visual impaired people by fusing non-Gaussian distributed probabilistic maps with GNSS.

Apart from these applications that combine GNSS PVT estimates with other sensors, particle filters are also applied within the Bayesian Direct Position Estimation (BDPE) [14,15] that aims to estimate the PVT of a GNSS receiver directly from the signal. This promising direct positioning and signal processing approach increases the sensitivity in PVT tracking by $10\;log_{10}$(N)-dB, where N is the number of used GNSS signals [16]. Thus, this method was investigated in different GNSS related topics like GNSS signal acquisition performance in [17,18,19], improved positioning accuracy when fusing GNSS with visual information in [20] or improving the robustness of position and time estimation during jamming and meaconing attacks in [21,22]. The first real-time capable implementation of BDPE into a commercial software based GNSS receiver was shown in [23], even if suboptimal but computational efficient algorithms and limitations in number of tracked GNSS signals have been made as a tradeoff to achieve real-time performance.

The previously mentioned work presents the concept of particle filters and their elements, performance and implementation [6,7,8,9]. Current research topics of particle filters in GNSS are given in [10,11,12,13]. The concept and first implementations of (B)DPE can be found in [14,15,16,17,18,19,20,21,22,23], but the optimal weight update for BDPE is still an open topic. One major aspect of BDPE is to perform an ideal weight update during incorporation of a new set of measurements. Therefore, the goal of this work is to derive an optimal particle weight update function for multiple GNSS signals, to propose a solution for numerical stability during the update steps and to analyze the method using representative scenarios.

This work starts with an explanation of the existing BDPE implementation [23] and illustrations in Section 2, which is followed by a detailed description of the implemented particle filter in Section 3. The existing implementation uses BDPE concepts presented in [8,14,16,24], the Line-of-Sight (LOS) projection elements of a Vector Tracking Loop (VTL) for steering the GNSS internal replica signal generators as shown in [25], the concept of highly efficient synthetically generated multi-correlator values (MC) as shown in [26] and ideas of [17,18] for mapping the correlation values to particles of a Particle Filter (PF). Section 4 shows the derivation of the optimal particle weight, which is based on a probabilistic description of the GNSS signal from [27], and Section 5 shows how the update can be performed under consideration of a finite precision of a computer. Section 6 shows that the optimal weight causes a very dense concentration of particles, which can lead to suboptimal behaviour of Bayesian filters, especially in the case of different residual user range errors coming, e.g., from orbit, satellite clock, multipath or ionosphere. Therefore, a solution is proposed in Section 7, which limits the theoretical high ranging accuracies by considering an additional nuisance parameter. The work is concluded with simulations and real-world data in Section 8.

## 2. BDPE Receiver Architecture

**Figure 1:**
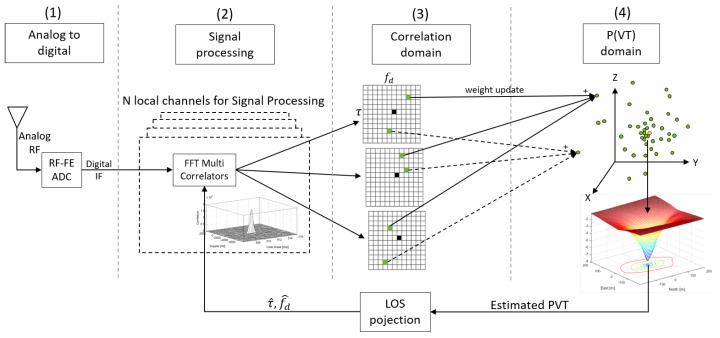
Bayesian Direct Position Estimation (BDPE) processing scheme as implemented in the software based Global Navigation Satellite System (GNSS) receiver; (**1**) refers to the analogue to digital conversion, the first stage of the receiver; (**2**) refers to the GNSS signal processing, which produces correlation values for each tracked channel as symbolically shown in (**3**). The correlation values are mapped and weighted to a particle cloud of a particle filter as shown in (**4**).

The concept of BDPE has been implemented into the SX3 [28], a commercial software-based GNSS receiver (IFEN GmbH, Poing, Germany). The implementation follows the idea to make maximum reuse of existing, well proven and computationally optimized GNSS receiver elements. The implemented receiver design is shown in Figure 1 and it consists of four blocks.

The first block (**1**) refers to the first stage of the GNSS signal processing. The Radio Frequency (RF) signal is gathered with a GNSS antenna. Afterwards, the RF signal is down-converted to an Intermediate Frequency (IF). Then, the analogue IF signal is converted into a digital signal by an Analogue to Digital Converter (ADC). Finally, the digital and to IF down-converted sample stream is transmitted via USB to a PC, where the whole GNSS receiver processing is performed.

The second step in the processing sequence is the signal correlation, indicated with (**2**) in Figure 1. At this stage, the receiver is configured to act in the same way as a vector tracking receiver. For each of the *N* GNSS signals, a complex-valued replica signal is generated using the best estimate $\hat{f_{d}}$ and $\hat{\tau }$, at the Doppler and code phase. These estimates come from the latest PVT estimate (cf. step **4**) after applying a LOS projection. Thus, there are Doppler and code phase estimates for each of the *N* channels. The signal replica is then correlated with the incoming signal to produce a new time series of correlation values. This is done for a wide range of code phase offsets, not only for Early, Prompt and Late, as it is typically done in GNSS receivers. Thereafter, a post-correlation Fast Fourier Transform (FFT) is applied to this complex valued correlation values in order to obtain synthetically generated correlation values in the Doppler direction. The resulting grid of correlation values in code phase and Doppler direction are called synthetically generated Multi-Correlation (MC) values (cf. step **3**). These maps contain now correlation values for a range of Doppler bins and code phase offsets. The MC processing performs a navigation data bit wipeoff which also allows for long coherent integration times. The MC maps are centered at the old fed back Doppler $\hat{f_{d}}$ and code phase $\hat{\tau }$, symbolically indicated with the black square. In the next step, each green PVT particle of a particle filter in the PVT domain (**4**) is mapped to its corresponding correlation value in (**3**). This is done for each channel *N* and using a sinc interpolation in both directions, code phase and Doppler. Based on the fact that the correlation values are coupled via the geometric relationship of its PVT particle, it is allowed to sum them up, the basic idea of DPE. The correlation values are used as input to determine the weight change of a particle, a crucial step in a Bayesian filtering approach, which is discussed in detail within this work. Using synthetically generated correlation values as input to the Bayes filter is a valid approach for DPE because it was proven in [26] that these synthetically generated correlation values represent mathematically the exact value of a real correlator. This approach mimics the direct correlation and it is computationally very efficient.

**Figure 2:**
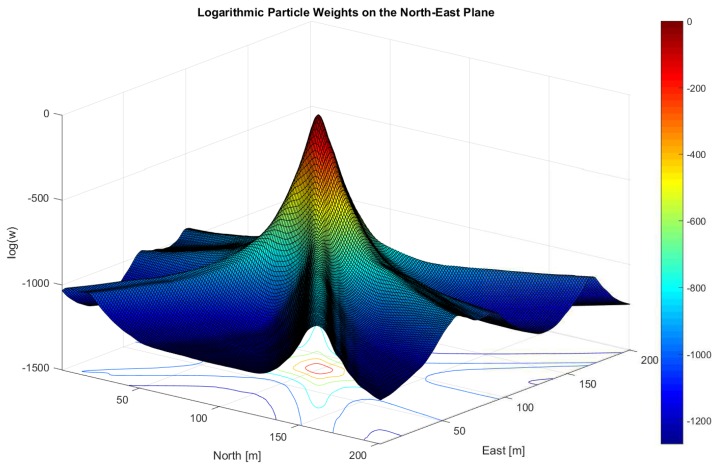
The normalized logarithmic particle weights ${\tilde{w}}_{k}^{i}$ from Equation (35), assuming a uniform distribution from the previous epoch, i.e., ${\tilde{w}}_{k-1}^{i}=0$ and using the optimal particle weight update from Equation (34) are shown. The particles are equidistantly distributed over a grid in the northeast plane. The lines through the plot correspond to the weighted correlation function in the position domain and thus refer to a GNSS signal. In a proper case (correct user velocity, clock error and drift), the lines overlap at a distinct point in the position domain, which is in this case the northeast plane. The resulting peak represents the probability of the 2D position. Note that the plotted weights are normalized and in the logarithmic scale, thus the peak has the maximum value of 0. The coherent integration time for this plot was set to $T_{coh}=2$ ms. The processed data refers to open sky. It was recorded at latitude LAT = 47.06446263 deg, longitude LON = 15.40777110 deg on the rooftop of Reininghausstraße 13a, Graz, Austria.

**Figure 3:**
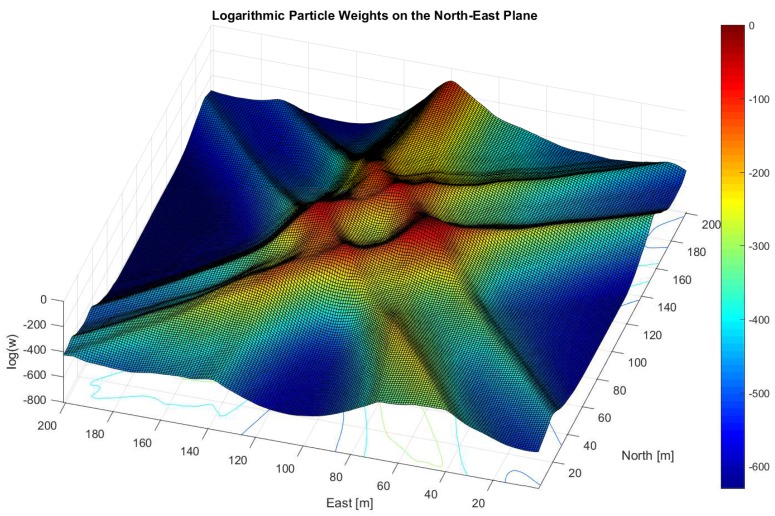
This plot depicts a case when clock error is improperly aligned. The GNSS signals do not overlap at a distinct position in the northeast plane. The plot shows the normalized logarithmic particle weights ${\tilde{w}}_{k}^{i}$ from Equation (35), assuming a uniform distribution from the previous epoch, i.e., ${\tilde{w}}_{k-1}^{i}=0$ and using the optimal particle weight from Equation (34). The particles are equidistantly distributed over a grid. The lines through the plot correspond to the weighted correlation function in the position domain and thus refer to a distinct GNSS signal. The lines look very broad even after the weight update, which comes from the logarithmic scale given by $\log(w_{k}^{i})$. The coherent integration time for this plot was set to $T_{coh}=2$ ms. The processed data refers to open sky. It was recorded at LAT = 47.06446263 deg, LON = 15.40777110 deg on the rooftop of Reininghausstraße 13a, Graz, Austria.

The last block (**4**) in Figure 1 refers to the navigation processor and thus to the implemented particle filter. There exist various types of particle filters—for example, Sampling Importance Resampling (SIR), Auxiliary SIR (ASIR) or Regularized Particle Filter (RPF). The details of these filters are not further discussed in this work, but all of them rely on a proper particle weight update. The particle weight update function determines the weight change of each PVT particle and takes the MC values as input. Afterwards, the final PVT solution (yellow circle in **4**) is computed by constructing a probability density function using the particle weights and calculating the final PVT solution using a weighted mean over all particles. To illustrate the implementation, Figure 2 depicts the normalized logarithmic particle weights ${\tilde{w}}_{k}^{i}$ from Equation (35) using the optimal particle weight update from Equation (34) but without information from the previous epoch ${\tilde{w}}_{k-1}^{i}=0$. Thus, there is no influence from the past and the figure shows the results from one epoch only. In contrast to it, Figure 3 shows an improper aligned clock error where the GNSS signals do not overlap in the position domain.

In BDPE, the implemented Bayesian filter plays an important role as it estimates the PVT solution from all available measurements. The following chapter discusses in detail the Bayesian framework and its role in the concept of DPE.

## 3. Particle Filter

This chapter discusses briefly the basic principles of a particle filter in order to cover the complete framework of the investigated weight update step, which follows in the next chapters. Particle filters belong to the group of non-parametric Bayesian filters [13] and thus allow for dealing with nonlinear and non-Gaussian models [8]. In order to estimate the state of a dynamic system, two filter steps are required, *Prediction* and *Update*. The prediction step is based on the Chapman–Kolmogorov equation, which propagates the previous state $x_{k-1}$ to the prior state $x_{k}$ by

(1) $$p\left(x_{k}|z_{1:k-1}\right)=\int p\left(x_{k}|x_{k-1}\right)p\left(x_{k-1}|z_{1:k-1}\right)dx_{k-1},$$

using the system model

(2) $$x_{k}=f_{k}\left(x_{k-1},v_{k-1}\right),$$

where z is a set of all available measurements, and $f_{k}\left(\cdot \right)$ is a possibly nonlinear function with the corresponding process noise $v_{k-1}$, at epoch *k*. The process noise can be interpreted as the uncertainty of the system model, which is accounted for when propagating the system state. The incorporation of new information (measurements) is performed in the update step, which is described by the Bayes rule

(3) $$p\left(x_{k}|z_{1:k}\right)=\frac{p\left(z_{k}|x_{k}\right)p\left(x_{k}|z_{1:k-1}\right)}{p(z_{k}|z_{1:k-1})}$$

with the normalization constant

(4) $$p\left(z_{k}|z_{1:k-1}\right)=\int p\left(z_{k}|x_{k}\right)p\left(x_{k}|z_{1:k-1}\right)dx_{k}.$$

The update incorporates the new measurement $z_{k}$ by using the measurement model, which relates the measurement $z_{k}$ to the states $x_{k}$ as

(5) $$z_{k}=h_{k}\left(x_{k},n_{k}\right),$$

where $h_{k}\left(\cdot \right)$ is a possibly nonlinear function and where $n_{k}$ denotes the measurement noise, which describes the uncertainty of the measurement. Now, the basic idea of a particle filter is to recursively approximate the posterior probability function. If considering only the case that a filtered estimate is required at each time step, a simplified discrete approximation of the posterior probability density function (PDF) without dependency on the complete history can be written as

(6) $$p\left(x_{k}|z_{1:k}\right)\approx \sum_{i=1}^{N_{s}}w_{k}^{i}\delta \left(x_{k}-x_{k}^{i}\right)$$

with

(7) $$w_{k}^{i}\propto w_{k-1}^{i}\frac{p\left(z_{k}|x_{k}^{i}\right)p\left(x_{k}^{i}|x_{k-1}^{i}\right)}{q(x_{k}^{i}|x_{k-1}^{i},z_{k})},$$

where the posterior probability only depends on the previous state at epoch k−1 and current measurement at epoch *k*. The approximation of the PDF in (6) is based on a set of support points $\left\{x_{k}^{i},i=1,\ldots ,N_{s}\right\}$ with associated weights $w_{k}^{i}$, where the weights are normalized such that $\sum_{i=1}^{N_{s}}w_{k}^{i}=1$. The δ(·) in (6) denotes the Dirac delta measure. It is a crucial step of the particle filter to obtain the current epoch weight $w_{k}^{i}$, which depends proportionally on the previous epoch’s weight $w_{k-1}^{i}$ which is propagated in time using by $p(x_{k}^{i}|x_{k-1}^{i})$ through (2), updated with new information by $p(z_{k}|x_{k}^{i})$ using the relation in (5) and normalized by the the importance density $q(x_{k}^{i}|x_{k-1}^{i},z_{k})$. The importance density q(·) defines the probability distribution of the support points, which is chosen in the presented implementation to be Gaussian distributed with an approximate mean of the truth in the very first step. The choice of q(·) is an important design step of a particle filter and one common approach is to choose the prior PDF such as

(8) $$q\left(x_{k}|x_{k-1}^{i},z_{k}\right)=p\left(x_{k}|x_{k-1}^{i}\right),$$

which leads to the simple weight update equation

(9) $$w_{k}^{i}\propto w_{k-1}^{i}p\left(z_{k}|x_{k}^{i}\right).$$

The realization of a particle filter using (9) is known as a Bootstrap filter. The concept of a Bootstrap filter is also implemented within this work. To be able to update the weights optimally, a probabilistic measurement model for $p(z_{k}|x_{k}^{i})$ in (9) with dependency on the prior state must be available. In the proposed approach, the state vector $x_{k}$ at current epoch *k* is an 8-element vector containing the GNSS receiver position, velocity and time, where the position and velocity take three elements each and time two elements refer to the receiver clock error and receiver clock drift. The measurements $z_{k}$ at current epoch *k* refer to a vector containing the raw GNSS samples. In order to update the particle weight, BDPE directly maps the PVT at the particles to the raw signal samples, which contains a superposition of all GNSS signals. Thus, a probabilistic description of the samples dependent on multiple GNSS signals must be available. Such a probabilistic model exists in literature (see, e.g., (5.9) of [27]) for a single GNSS signal and is given as

(10) $$p\left(s|A,\tau ,\omega ,\phi \right)=\frac{1}{{\left(2\pi \right)}^{L}}\exp\left\{-\frac{1}{2}\sum_{\mu =1}^{L}{\left|s_{\mu }-\underset{\mathrm{Signal}\;\mathrm{Model}}{\underset{︸}{Ac\left(t_{\mu }-\tau \right)\exp\left\{i\omega t_{\mu }-i\phi \right\}}}\right|}^{2}\right\},$$

where the vector $s\in C^{L}$ contains all signal samples $s_{\mu },\mu \in \left\{1,\ldots ,L\right\}$, which depend on the signal model parameters, the real valued signal amplitude $A\in R^{+}$, the time delay τ∈R, the Doppler frequency ω∈R, the uniformly distributed carrier phase ϕ∈[0,2π[ and *i* the imaginary number. The term $c\left(t_{\mu }-\tau \right)\in \left[-1,1\right]$ refers to the PRN code and the term $\exp\{i\omega t_{\mu }-i\phi \}$ to the signal carrier. The parameter $t_{\mu }\in R$ defines the time at sample index μ and depends on the sampling frequency $f_{s}\in R$ such as $t_{\mu }=\mu /f_{s}$. The probability distribution $p(z_{k}|x_{k}^{i})$ in the weight update Equation (9) can be related to the sample distribution in (10), where $z_{k}$ equals the measured sample vector such as $s=z_{k}$, where $t_{\mu =1}$ corresponds to the time of the first sample in epoch *k* and $t_{\mu =L}$ to the time of the last sample in epoch *k*. The parameters A,τ,ω,ϕ for a single replica can directly be related to the PVT state $z_{k}$, respectively, to the state of each PVT particle. The state $z_{k}$ is defined as

(11) $$z_{k}=\left[\begin{array}{c} r_{rx}\\ v_{rx}\\ t_{rx} \end{array}\right]=\left[\begin{array}{c} {\left[x,y,z\right]}^{T}\\ {}[v_{x},v_{y},v_{z}{]}^{T}\\ {}[t_{rxClkErr},t_{rxClkDrift}{]}^{T} \end{array}\right],$$

where the three-dimensional position $r_{rx}$ and three-dimensional velocity $v_{rx}$ relate to the Earth Centered Earth Fixed (ECEF) reference frame. The time vector $t_{rx}$ contains the GNSS receiver clock error $t_{rxClkErr}$ and clock drift $t_{rxClkDrift}$. The replica signal code phase $\tau_{n}$ and Doppler $\omega_{n}$ for a single signal of satellite *n* are related to $z_{k}$ using

(12) $$\tau_{n}=\frac{\overset{\mathrm{geom}.\;\mathrm{distance}}{\overset{︷}{\left|\right|r_{sat,n}-r_{rx}\left|\right|}}}{c}+t_{rxClkErr}+T_{n}+I_{n},$$

(13) $$\omega_{n}=-(\underset{\;\mathrm{rel}.\;\mathrm{velocity}\;}{\underset{︸}{v_{sat,n}-v_{rx}{)}^{T}}}\underset{\mathrm{unit}\;\mathrm{vector}\;u_{i}\;}{\underset{︸}{\frac{r_{sat,n}-r_{rx}}{\left|\right|r_{sat,n}-r_{rx}\left|\right|}}}\frac{f_{c}}{c}+t_{rxClkDrift}f_{c},$$

where $r_{sat,n}$ and $v_{sat,n}$ correspond to the position and velocity of satellite *n*, *c* to the speed of light, $T_{n}$ to the tropospheric delay, $I_{n}$ to the satellite and frequency dependent ionospheric delay and $f_{c}$ to the carrier frequency. These relationships assume that the receiver position is known at epoch *k* and thus the ephemeris of the satellite *n* for the generated replica signal needs to be available. In a simplified simulated setup without ionosphere, troposphere and other errors, the terms $I_{n}$ and $T_{n}$ may reduce to zero and can be neglected. The simulations which are performed within this work do not consider any geometric relationship, troposphere and ionosphere for analysis, but the real-world examples fully consider the geometric relationship as well as the ionospheric and tropospheric terms.

Basically the amplitude *A* and carrier phase ϕ can also be estimated from the particle filter, but this further increases the dimensionality of the estimation problem form currently 8 to 10 dimensions. To avoid a further increase in dimensionality and thus computational complexity and be able to take the synthetically generated multi-correlation values which only depend on code-phase τ and Doppler ω, the proposed approach is to make the probability function independent of the signal amplitude *A* and carrier phase ϕ, which is discussed in the next chapter.

## 4. Derivation of the Optimal Particle Weight for Multiple GNSS Signals

Basically, the goal of the derivation is to retrive a formulation for an optimal particle weight in dependence of an abitrary number of used GNSS signals dependent on the code phase τ and Doppler ω only. Thus, the following equations show the extension of the probabilistic model from single to multiple GNSS signals in Equation (17) with dependency on *A*, τ, ω, ϕ, in Equation (24) with dependency on *A*, τ, ω and in Equation (27) with dependency on τ, ω.

Under the assumption that the carrier phase is uniformly distributed over 0 and 2π, it can be integrated out as shown in [27] to

(14) $$\begin{array}{ccc} p(s|A,\tau ,\omega ) & = & \frac{1}{{\left(2\pi \right)}^{L}}\int_{\phi =0}^{2\pi }\exp\left\{-\frac{1}{2}\sum_{\mu =1}^{L}{\left|s_{\mu }-Ac\left(t_{\mu }-\tau \right)\exp\left\{i\omega t_{\mu }-i\phi \right\}\right|}^{2}\right\}d\phi \\ & = & \frac{1}{{\left(2\pi \right)}^{L}}\exp\left\{-\frac{1}{2}\sum_{\mu =1}^{L}{\left|s_{\mu }\right|}^{2}\right\}\exp\left\{-\frac{LA^{2}}{2}\right\}I_{0}\left(A|P\left(\tau ,\omega \right)\right|\sqrt{L}) \end{array}$$

with the definition of the correlator value $P_{n}\left(\tau ,\omega \right)$ as

(15) $$P_{n}\left(\tau ,\omega \right)=\frac{1}{\sqrt{L}}\sum_{\mu =1}^{L}{\bar{s}}_{\mu }c_{n}\left(t_{\mu }-\tau \right)\exp\left\{-i\omega t_{\mu }\right\},$$

where the correlation value $P_{n}\left(\tau ,\omega \right)$ is taken from the multi-correlation maps as shown in Figure 1 and $I_{0}$ referes to the Bessel function of the first kind and order zero. A similar equation for joint detection of weak GNSS signals can be found in [18]. In order to extend the model to multiple GNSS signals, let’s do this in the first step for two signals and find later a mathematical rule to extend it to multiple signals. The basic model for two signals is defined as

(16) $$\begin{array}{ccc} q_{8} & = & [A_{1},\tau_{1},\omega_{1},\phi_{1},A_{2},\tau_{2},\omega_{2},\phi_{2}], \end{array}$$

(17) $$\begin{array}{ccc} p(s|q_{8}) & = & \frac{1}{{\left(2\pi \right)}^{L}}\exp\left\{\underset{m}{\underset{︸}{-\frac{1}{2}\sum_{\mu =1}^{L}|s_{\mu }-\left[\overset{r_{1}}{\overset{︷}{A_{1}c_{1}\left(t_{\mu }-\tau_{1}\right)\exp\left\{i\omega_{1}t_{\mu }-i\phi_{1}\right\}}}+\overset{r_{2}}{\overset{︷}{A_{2}c_{2}\left(t_{\mu }-\tau_{2}\right)\exp\left\{i\omega_{2}t_{\mu }-i\phi \right\}}}\right]{|}^{2}}}\right\} \end{array},$$

and with the definition of $\left|z\right|=\sqrt{z\bar{z}}$, ${\left|z\right|}^{2}=z\bar{z}$ and the redefinition of the replica signals $r_{1}$ and $r_{2}$, *m* can be expanded to

(18)m=−12∑μ=1Lsμsμ¯−sμ[r1+r2]¯−sμ¯[r1+r2]+[r1+r2][r1+r2]¯(19)=−12∑μ=1Lsμsμ¯−sμr1¯−sμr2¯−sμ¯r1−sμ¯r2+r1r1¯+r2r1¯+r1r2¯+r2r2¯(20)=−12∑μ=1L(|sμ|2+|r1|2−2Re(sμ¯r1)+|r2|2−2Re(sμ¯r2)+2Re(r1¯r2)︸crosscorrelationterm≈0︷newtermsdueto2ndsignal.

With $|r_{n}{|}^{2}=A_{n}^{2}{\left|c\left(t_{\mu }-\tau_{n}\right)\right|}^{2}$ and neglecting the cross-correlation terms leads to

(21) $$\begin{array}{lll} m & = & -\frac{1}{2}\sum_{\mu =1}^{L}\left(\right|s_{\mu }{|}^{2}+A_{1}^{2}{\left|c_{1}\left(t_{\mu }-\tau_{1}\right)\right|}^{2}-2Re\left(\bar{s_{\mu }}A_{1}c_{1}\left(t_{\mu }-\tau_{1}\right)\exp\left\{i\omega_{1}t_{\mu }-i\phi_{1}\right\}\right)\\ & & +A_{2}^{2}{\left|c_{2}\left(t_{\mu }-\tau_{2}\right)\right|}^{2}-2Re\left(\bar{s_{\mu }}A_{2}c_{2}\left(t_{\mu }-\tau_{2}\right)\exp\left\{i\omega_{2}t_{\mu }-i\phi_{2}\right\}\right)). \end{array}$$

Insert Equation (21) in Equation (17), consider that the sum of the squared PRN code leads to $\sum_{\mu =1}^{L}{\left|c\left(t_{\mu }-\tau_{2}\right)\right|}^{2}=L$ and realigning the terms to integrate out $\phi_{1}$ and $\phi_{2}$ leads to

(22)q6=[A1,τ1,ω1,A2,τ2,ω2]p(s|q6)=1(2π)Lexp−12∑μ=1L|sμ|2,(23)1(2π)∫ϕ1=02πexp−LA122expA1∑μ=1LRe(sμ¯c1(tμ−τ1)exp{iω1tμ−iϕ1dϕ11(2π)∫ϕ2=02πexp−LA222expA2∑μ=1LRe(sμ¯c2(tμ−τ2)exp{iω2tμ−iϕ2dϕ2=1(2π)Lexp−12∑μ=1L|sμ|2(24)exp−LA122I0(A1|P1(τ1,ω1)1|L)exp−LA222I0(A2|P2(τ2,ω2)2|L).

It can be shown in Equation (24) that the extension to multiple GNSS follows the same structure as shown in Equation (14) for a single GNSS signal, if the orthogonal cross correlation terms are neglected as shown in Equation (20).

Solving an integral of type $\int_{0}^{\infty }\exp\left\{-\frac{x^{2}}{c}\right\}I_{0}\left(xp\right)dx$ was performed with the help of Wolfram Mathematica (Version 11.3, Wolfram Research Inc., Champaign, IL, USA) [29], which helps to integrate out the amplitude as

p(s|τ1,ω1,τ2,ω2)=1(2π)Lexp−12∑μ=1L|sμ|2(25)∫A1=0∞exp−LA122I0(A1|P1(τ1,ω1)1|L)dA1∫A2=0∞exp−LA222I0(A2|P2(τ2,ω2)2|L)dA2=1(2π)Lexp−12∑μ=1L|sμ|2(26)π2Lexp|P1(τ1,ω1)|24I0|P1(τ1,ω1)|24π2Lexp|P2(τ2,ω2)|24I0|P2(τ2,ω2)|24.

With the assumption of using Gaussian distributed complex valued samples $s_{\mu }$ whose real and imaginary parts are each of variance one and zero mean, the sum over the absolute squared samples approximates to $L\approx \frac{1}{2}\sum_{\mu =1}^{L}{\left|s_{\mu }\right|}^{2}$. This approximation can be made if the GNSS signal amplitude is significantly smaller than the noise. This is generally the case for GNSS signals when considering receivers operated on the ground [1]. With this approximation, Equation (26) can be rewritten for *N* GNSS signals as

(27) $$\begin{array}{c} p\left(s|\tau ,\omega \right)=\frac{1}{{\left(2\pi \right)}^{L}}\exp\left\{-L\right\}N\sqrt{\frac{\pi }{2L}}\prod_{n=1}^{N}\exp\left\{\frac{|P_{n}\left(\tau_{n},\omega_{n}\right){|}^{2}}{4}\right\}I_{0}\left(\frac{|P_{n}\left(\tau_{n},\omega_{n}\right){|}^{2}}{4}\right). \end{array}$$

Equation (27) defines the probability function $p(z_{k}|x_{k}^{i})$ for an arbitrary number *N* GNSS signals in order to obtain $w_{k}^{i}$ in Equation (9). Note that the code-phase and Dopplers changed now to vectors $\tau \in R^{N}$ and $\omega \in R^{N}$. However, with this equation, the weight can not be evaluated directly due to finite precision effects. In particular, the terms $\exp\left\{\frac{|P_{n}\left(\tau_{n},\omega_{n}\right){|}^{2}}{4}\right\}$ and $I_{0}\left(\frac{|P_{n}\left(\tau_{n},\omega_{n}\right){|}^{2}}{4}\right)$ use a typically ’large’ correlation value *P*, which causes a numerical problem. The common maximum value that can be stored on 64-bit platforms is a double precision floating point value (not considering dedicated floating point libraries). The maximum value is limited to 1.7E ± 308 (15 digits) [30]. Considering the term $\exp\left\{\frac{|P_{n}\left(\tau_{n},\omega_{n}\right){|}^{2}}{4}\right\}$ in Equation (27), the numerical limits are reached for a correlation value $P_{n}\left(\tau_{n},\omega_{n}\right)>53.28$ approximately. A solution to overcome the effect of limited digital precision is proposed in the next chapter.

## 5. Logarithmic Weight Update

When performing the particle weight update, the numerical values can be too large to be evaluated by a computer. The numerical issues at the weight update when evaluating Equation (27) can be solved by shifting the equations to the logarithmic scale and performing an additional normalization step. As a first step, let

(28) $$\begin{array}{c} I_{\alpha }\left(z\right)\approx \frac{e^{z}}{\sqrt{2\pi z}}\left(1-\frac{4\alpha^{2}-1}{8z}+\frac{\left(4\alpha^{2}-1\right)\left(4\alpha^{2}-9\right)}{2!{\left(8z\right)}^{2}}+\ldots \right) \end{array}$$

be an approximation of the Bessel function of first kind where only the first term is used to approximate the function with order zero as

(29) $$\begin{array}{c} I_{0}\left(z\right)\approx \frac{e^{z}}{\sqrt{2\pi z}}. \end{array}$$

With Equation (29), Equation (27) can be rewritten to

(30) $$\begin{array}{c} p\left(s|\tau ,\omega \right)\approx \underset{H_{const}}{\underset{︸}{\frac{1}{{\left(2\pi \right)}^{L}}\exp\left\{-L\right\}N\sqrt{\frac{\pi }{2L}}}}\prod_{n=1}^{N}\left(\frac{\exp\left\{\frac{|P_{n}\left(\tau_{n},\omega_{n}\right){|}^{2}}{2}\right\}}{\sqrt{2\pi \frac{|P_{n}\left(\tau_{n},\omega_{n}\right){|}^{2}}{4}}}\right). \end{array}$$

Based on the reason that a later introduced normalization step is performed in Equation (35), the constant term $H_{const}$ can be neglected. With that, the weight update Equation in (9) can be expressed as

(31) $$w_{k}^{i}\propto w_{k-1}^{i}\prod_{n=1}^{N}\left(\frac{\exp\left\{\frac{|P_{n}\left(\tau_{n},\omega_{n}\right){|}^{2}}{2}\right\}}{\sqrt{2\pi \frac{|P_{n}\left(\tau_{n},\omega_{n}\right){|}^{2}}{4}}}\right).$$

Let us define ${\tilde{w}}_{k}^{i}=\log\left(w_{k}^{i}\right)$ and ${\tilde{w}}_{k-1}^{i}=\log\left(w_{k-1}^{i}\right)$; then, the update can be written in logarithmic scale as

(32) $$\begin{array}{c} {\tilde{w}}_{k}^{i}\propto {\tilde{w}}_{k-1}^{i}+\sum_{n=1}^{N}\left(\frac{|P_{n}\left(\tau_{n},\omega_{n}\right){|}^{2}}{2}-\log\left(\sqrt{2\pi \frac{|P_{n}\left(\tau_{n},\omega_{n}\right){|}^{2}}{4}}\right)\right). \end{array}$$

The weight update from Equation (9) can now be done in five steps from Equation (33) to Equation (37) using

(33) $$\begin{array}{ccc} {\tilde{w}}_{k-1}^{i} & = & \log(w_{k-1}^{i}), \end{array}$$

(34) $$\begin{array}{ccc} {\tilde{w}}_{k}^{i} & = & {\tilde{w}}_{k-1}^{i}+\sum_{n=1}^{N}\left(\frac{|P_{n}\left(\tau_{n},\omega_{n}\right){|}^{2}}{2}-\log\left(\sqrt{2\pi \frac{|P_{n}\left(\tau_{n},\omega_{n}\right){|}^{2}}{4}}\right)\right), \end{array}$$

(35) $$\begin{array}{ccc} {\tilde{w}}_{k}^{i} & = & {\tilde{w}}_{k}^{i}-\max\left({\tilde{w}}_{k}^{i}\right), \end{array}$$

(36) $$\begin{array}{ccc} w_{k}^{i} & = & \exp({\tilde{w}}_{k}^{i}), \end{array}$$

(37) $$\begin{array}{ccc} w_{k}^{i} & = & \frac{w_{k}^{i}}{\sum_{i=1}^{N_{s}}w_{k}^{i}}. \end{array}$$

This series of equations allows for performing the weight update within numerical boundaries (e.g., double precision) and can generally be used to implement the optimal particle weight update in any Bayesian filter working with correlator values.

## 6. Discussion of the Ranging Accuracy

**Figure 4:**
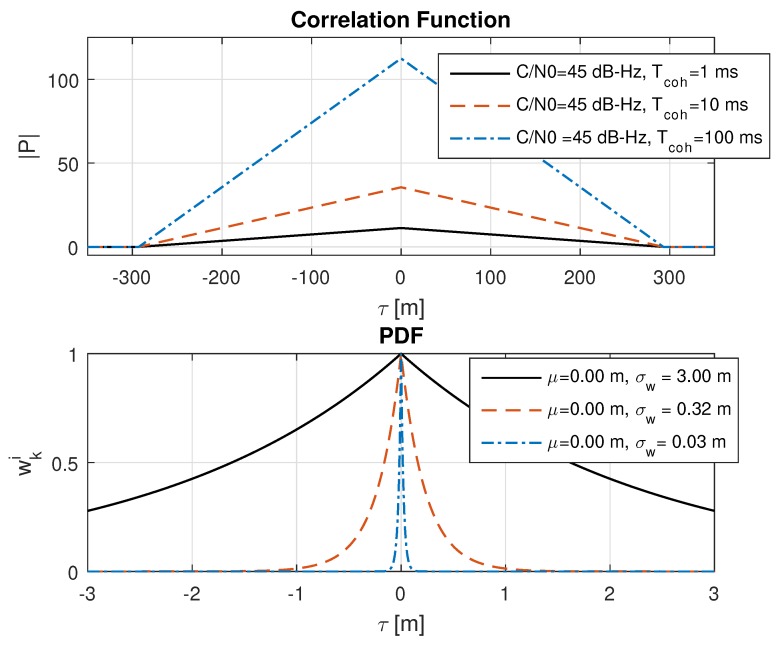
The plot is based on a MATLAB [31] simulation (Release 2015b, The MathWorks Inc., Natick, MA, USA). The upper plot shows the correlation function from Equation (15) for different coherent integration times at a C/N0 = 45 dB-Hz. The simulated signal was generated without noise (i.e., the C/N0 merely defines the correlation amplitude). The lower plot shows the corresponding probability function after one weight update Equation (36), for one signal N=1 and assuming a uniform distribution from the previous epoch, i.e., ${\tilde{w}}_{k-1}^{i}=0$ is considered in Equation (34). The statistics in the lower plot refer to the weighted mean μ and weighted standard deviation $\sigma_{w}$.

A further investigation determined that the probability distribution after the weight update from Equation (36) shows a very small variance in range, especially for strong GNSS signals and long integration times (e.g., larger than ten milliseconds). This is briefly illustrated in Figure 4. It can be seen that the amplitude of the correlation value has a significant impact on the variance of the probability function. For a typical open sky case at 45 to 50 dB-Hz and integration times of ≥10 ms, a weighted standard deviation of ≤0.32 m would be observed. This is caused by the large amplitude of the correlation function and small variations in the range cause already a significant variation in P(τ).

This behavior is expected and consistent with standard tracking theory and for example represented by the Cramér–Rao lower bound (CRLB) of the code pseudorange noise. However, the ranging accuracy described by the CRLB is never reached in practice, as multipath, residual orbit or satellite clock errors or residual ionospheric delays by far exceed the CRLB. Least-squares positioning algorithms or a Kalman filter (both working with code pseudoranges) can cope well with this increased ranging errors as the overall ranging accuracy is assumed to be unknown and is estimated from the code pseudorange residuals. Those algorithms only consider the relative accuracy variations between the different pseudoranges to define the relative weights, but do not account for a common accuracy scale factor.

However, for DPE, it is expected that this narrow probability distribution of the weights cause problems. First, grid based filters with fixed resolution and extension may not be able to cover the narrow probability distribution appropriately. Second, a residual code delay bias (multipath, orbit, clock or iono) may shift the probability density of one satellite with respect to another. Instead of overlapping the true PVT, the shifted functions may assume very small values (or even be zero due to the finite precision in the computer) and thus eliminate particles at the true PVT. The particle cloud of the particle filter would be severely concentrated around one maximum and it is expected that the filter may also have difficulties to track the PVT solution accurately in case the amplitudes of the different GNSS signals vary in time relative to each other.

Thus, a trade-off and an adaptive approach for the grid resolution, grid extension and computational power seem to be necessary to guarantee a good handling of the narrow probability functions. One could think of increasing the process noise to cope with the delay biases, but as the process noise is determined by the dynamic model, this is not a viable approach.

The next chapter proposes a method to overcome these problems by introducing an unknown delay bias as nuisance parameter.

## 7. Delay Bias as Gaussian Nuisance Parameter

This section describes an approach to handle user range uncertainties (due to e.g., multipath, orbit, clock and iono) within a DPE framework, which are much larger than the code noise. The idea is to introduce an unknown normally distributed bias in the signal model which reflects uncertainties in the user range. This artificially degrades the accuracy of the raw signal but better reflects the true measurement situation and avoids situations of non-overlapping probability functions.

One may argue that this delay bias degrades the overall positioning accuracy, but this argument is only true for an ideal situation of vanishing range uncertainties. For real situations, Equation (27) does not reflect the range uncertainties and thus is therefore only an approximate model of the signal samples. Overall, we think that the parameter can be justified and its introduction produces more meaningful probability density function compared to the ideal case.

Basically, this bias can be applied at code phase τ and Doppler $f_{d}$, referred to as code delay bias Δτ and Doppler bias $\Delta f_{d}$. This work discusses and analyses the introduction of a code delay bias Δτ to the code delay τ→τ+Δτ, but the procedure for the Doppler bias is basically the same. The code delay bias Δτ should express orbit errors, ionospheric errors, clock errors and other modeling errors. It is assumed that these errors follow a normal distribution such that

(38) $$\begin{array}{ccc} \Delta \tau & ∼ & N(0,\sigma_{\Delta \tau }^{2}), \end{array}$$

(39) $$\begin{array}{ccc} p(\Delta \tau ) & = & \frac{1}{\sqrt{2\pi }\sigma_{\Delta \tau }}\exp\left\{-\frac{1}{2}\frac{\Delta \tau^{2}}{\sigma_{\Delta \tau }^{2}}\right\}. \end{array}$$

Considering for now only one signal and applying the delay bias from Equation (39) to Equation (26) leads to

(40) $$\begin{array}{ccc} p(s|\tau ,\omega ) & = & \int_{\Delta \tau =-\infty }^{\infty }p\left(\Delta \tau \right)p\left(s|\tau +\Delta \tau ,\omega \right)d\Delta \tau . \end{array}$$

As this integral cannot be further simplified analytically, we target an approximate evaluation at selected grid points $\Delta \tau_{k}$. Neglecting a normalization factor Equation (40) can be expressed as

(41) $$\begin{array}{ccc} p(s|\tau ,\omega ) & \approx & \sum_{k=1}^{K}p\left(\Delta \tau_{k}\right)p\left(s|\tau +\Delta \tau_{k},\omega \right). \end{array}$$

The grid points $\Delta \tau_{k}$ should be chosen in a way to cover a significant part of $N(0,\sigma_{\Delta \tau }^{2})$ from Equation (38) with a reasonable resolution. In this work, we choose a range of $\pm 3\sigma_{\Delta \tau }$ with a resolution of 0.01 m. Under the assumption that the introduced code delay bias follows the same distribution for both signals, Equation (41) can be rewritten to

(42) $$\begin{array}{ccc} p(s|\tau_{1},\omega_{1},\tau_{2},\omega_{2}) & = & \frac{1}{{\left(2\pi \right)}^{L}}\exp\left\{-\frac{1}{2}\sum_{\mu =1}^{L}{\left|s_{\mu }\right|}^{2}\right\}\\ & & \sum_{k=1}^{K}p\left(\Delta \tau_{k}\right)\sqrt{\frac{\pi }{2L}}\exp\left\{\frac{|P_{1}\left(\tau_{1}+\Delta \tau_{k},\omega_{1}\right){|}^{2}}{4}\right\}I_{0}\left(\frac{|P_{1}\left(\tau_{1}+\Delta \tau_{k},\omega_{1}\right){|}^{2}}{4}\right)\\ & & \sum_{k=1}^{K}p\left(\Delta \tau_{k}\right)\sqrt{\frac{\pi }{2L}}\exp\left\{\frac{|P_{2}\left(\tau_{2}+\Delta \tau_{k},\omega_{2}\right){|}^{2}}{4}\right\}I_{0}\left(\frac{|P_{2}\left(\tau_{2}+\Delta \tau_{k},\omega_{2}\right){|}^{2}}{4}\right). \end{array}$$

Applying the same simplification steps as was done to obtain Equations (27) and (30) leads to a weight update equation similar to Equation (31) with

(43) $$w_{k}^{i}\propto w_{k-1}^{i}\prod_{n=1}^{N}\left\{\sum_{k=1}^{K}\underset{c_{k}}{\underset{︸}{p\left(\Delta \tau_{k}\right)\frac{\exp\left\{\frac{|P_{n}\left(\tau_{n}+\Delta \tau_{k},\omega_{n}\right){|}^{2}}{2}\right\}}{\sqrt{2\pi \frac{|P_{n}\left(\tau_{n}+\Delta \tau_{k},\omega_{n}\right){|}^{2}}{4}}}}}\right\}.$$

Equation (43) is also numerically difficult to evaluate due to the large exponential. The sum over the grid points does not allow directly shifting the equation to the logarithmic domain in order to get rid of the exponential, thus some reformulation is necessary. Let us define

(44) $$\begin{array}{ccc} a_{k} & = & \frac{p(\Delta \tau_{k})}{\sqrt{2\pi \frac{|P_{n}\left(\tau_{n}+\Delta \tau_{k},\omega_{n}\right){|}^{2}}{4}}}, \end{array}$$

(45) $$\begin{array}{ccc} b_{k} & = & \frac{|P_{n}\left(\tau_{n}+\Delta \tau_{k},\omega_{n}\right){|}^{2}}{2}. \end{array}$$

Then, the inner term of the sum can be rewritten for K=2 to

(46) $$\sum_{k=1}^{2}c_{k}=a_{1}e^{b_{1}}+a_{2}e^{b_{2}}=a_{1}e^{b_{1}}\left(1+e^{b_{2}-b_{1}}\frac{a_{2}}{a_{1}}\right),$$

which can be written in a general form as

(47) $$\begin{array}{ccc} \sum_{k=1}^{K}c_{k} & = & a_{1}e^{b_{1}}\left(1+\sum_{k=2}^{K}\underset{\;<1\;\mathrm{if}\;b_{1,\max}\;}{\underset{︸}{e^{b_{k}-b_{1}}}}\frac{a_{k}}{a_{1}}\right). \end{array}$$

When selecting $b_{1}$ to be the maximum, the exponential term in the sum of Equation (47) remains <1. Applying the logarithm now leads to

(48) $$\begin{array}{ccc} \log\left(\sum_{k=1}^{K}c_{k}\right) & = & \log\left(a_{1}\right)+b_{1}+\log\left(1+\sum_{k=2}^{K}e^{b_{k}-b_{1}}\frac{a_{k}}{a_{1}}\right), \end{array}$$

which can be well evaluated numerically. Using this in Equation (43) and applying the logarithm leads to the new logarithmic weight update equation

(49)w˜ki=w˜k−1i+∑n=1Nlog(a1)+b1+log1+∑k=2Kebk−b1aka1(50)=w˜k−1i+∑n=1N[logp(Δτ1)2π|Pn(τn+Δτ1,ωn)|24+|Pn(τn+Δτ1,ωn)|22+log1+∑k=2Kexp|Pn(τn+Δτk,ωn)|22−|Pn(τn+Δτ1,ωn)|22p(Δτk)|Pn(τn+Δτ1,ωn)|p(Δτ1)|Pn(τn+Δτk,ωn)|].

Equation (50) can now be used instead of Equation (35) within the weight update sequence Equation (33) to Equation (37) to include the Gaussian delay bias. An additional sum over *K* grid points and a maximum search over $b_{k}$ needs to be evaluated for each signal *N*, which increases the computational complexity. It should, however, be noted that the computation needs to be performed only for each set of multicorrelator values for each satellite signal and then an interpolation can be performed to obtain the weight update for the individual particles. This avoids computation of Equation (50) for each particle.

## 8. Simulations and Real-World Results

The previous chapters presented the optimal weight update function in Equation (32) for DPE when using a Bayes filter and dicussed in Figure 4 the problem with the resulting narrow probability function. In Equation (50), a possible solution is proposed to overcome the presented issues. In order to understand the behaviour of the proposed weight update function, an analysis is performed for Equation (50) within this chapter for a single signal N=1 and assuming a uniform distribution from the previous epoch, i.e., ${\tilde{w}}_{k-1}^{i}=0$. All simulations have been performed completely with MATLAB, except the real-world scenarios, which take as input the correlation values from the GNSS software receiver. The MATLAB simulation performs a correlation with a simulated GNSS signal. In order to see clearly the impact of the different parameters, the simulated signal was generated without noise. All following plots show the correlation values |P| from Equation (15) and the resulting weight $w_{k}^{i}$ from Equation (36) when using Equation (50) as weight update function. For visualisation, Equation (36) is plotted instead of Equation (37). This is because of the normalized amplitude to 1, which makes a comparison more easy to visualize. It is noted that the analysis is based on the herein presented equations. The derived equations do not consider any present multipath signal, thus there is no estimation of multipath parameters nor a handling or mitigation of multipath.

The next subsections analyse the impact on the resulting probability function for

- different signal strengths,
- different code delay bias variances,
- constructive and destructive multipath,
- short, medium and far multipath,
- different multipath amplitudes,
- two significant real-world scenarios under open-sky and urban conditions.

### 8.1. Impact of Different Signal Strengths

**Figure 5:**
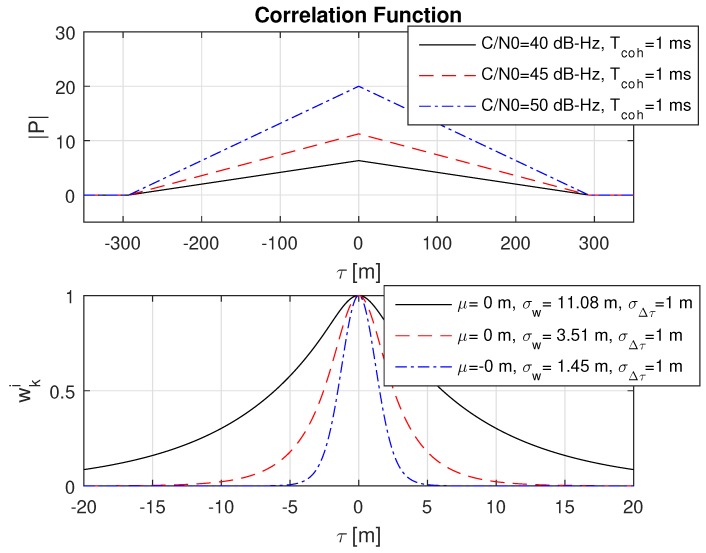
Impact of different GNSS signal amplitudes on the correlation values |P| and resulting probability function for a constant coherent integration time $T_{coh}$ and constant code delay bias standard devication $\sigma_{\Delta \tau }$. Higher signal strengths result in a smaller weighted standard deviation $\sigma_{w}$ of the probability function.

Basically, a higher GNSS signal amplitude increases the correlation value from Equation (15) and thus influences Equation (50). From this, it is expected that changes in amplitude do not shift the mean of the resulting probability function but influence the variance. This is obvious because a stronger received GNSS signal must result in a more accurate estimate, which is shown in Figure 5.

### 8.2. Impact of Different Code Delay Bias Variances

**Figure 6:**
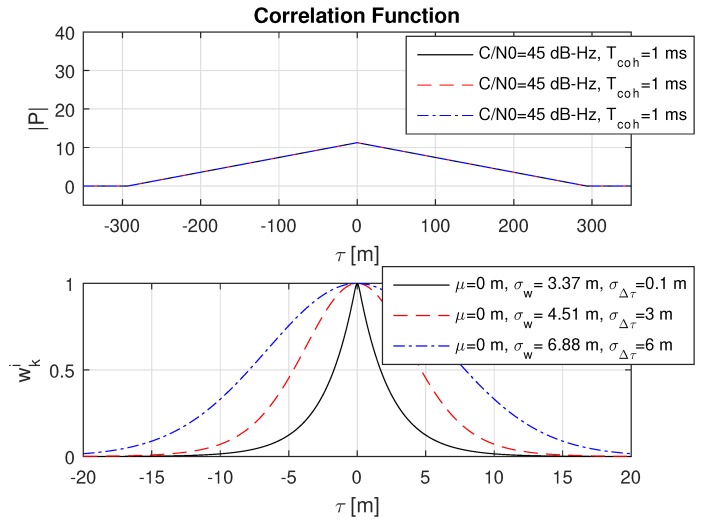
Impact of different code delay bias standard deviations $\sigma_{\Delta \tau }$ on the weights $w_{k}^{i}$ for a coherent integration time $T_{coh}=1$ ms. For the given conditions, only the weighted standard deviation $\sigma_{w}$ for $\sigma_{\Delta \tau }=6$ seems to approach the theoretical lower limit of $\sigma_{\Delta \tau }$. In particular, $\sigma_{\Delta \tau }=0.1$ can not be reached due to the influence of the low correlation time $T_{coh}$ at given *C*/N0.

**Figure 7:**
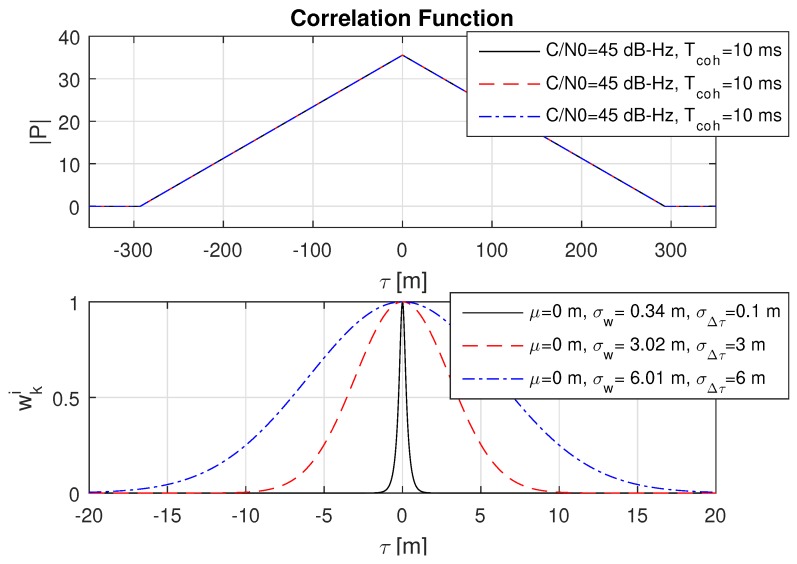
Impact of different code delay bias standard devications $\sigma_{\Delta \tau }$ on the weights $w_{k}^{i}$ for a coherent integration time $T_{coh}=10$ ms. It can be seen that $\sigma_{w}$ approaches the theoretical limits for $\sigma_{\Delta \tau }=3$ and $\sigma_{\Delta \tau }=6$.

**Figure 8:**
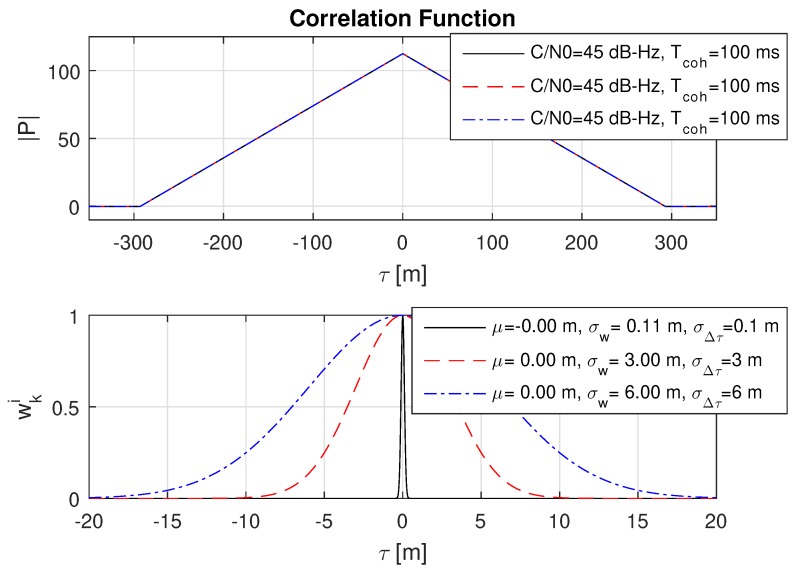
Impact of different code delay bias standard devications $\sigma_{\Delta \tau }$ on the weights $w_{k}^{i}$ for a coherent integration time $T_{coh}=100$ ms. It can be seen that $\sigma_{w}$ now approaches the theoretical limits for all $\sigma_{\Delta \tau }$. Note also the significantly changed amplitude on |P|.

Basically, the expected measurement accuracy $\sigma_{w}$ strongly depends on the amplitude of the correlation value |P| and thus on the coherent integration time $T_{coh}$ and the GNSS signal strength. However, with the introduction of a theoretical lower limit of the measurement accuracy in Equation (40) defined by $\sigma_{\Delta \tau }$, the estimated accuracy $\sigma_{w}$ must approach the lower limit $\sigma_{\Delta \tau }$ with an increasing correlation value |P|. This behaviour is shown in three consecutive plots in Figure 6, Figure 7 and Figure 8 with an increasing coherent integration time $T_{coh}=\left[1,10,100\right]$ ms. From this series, it is clearly visible that $\sigma_{w}$ also approaches a very small $\sigma_{\Delta \tau }=0.1$ m in the case of a long coherent integration time $T_{coh}=100$ ms.

The magnitude of $\sigma_{\Delta \tau }$ should cover residual user range errors (coming from orbit, satellite clock, multipath or ionospheric errors), which can be in the range of several meters.

### 8.3. Impact of Constructive and Destructive Multipath

**Figure 9:**
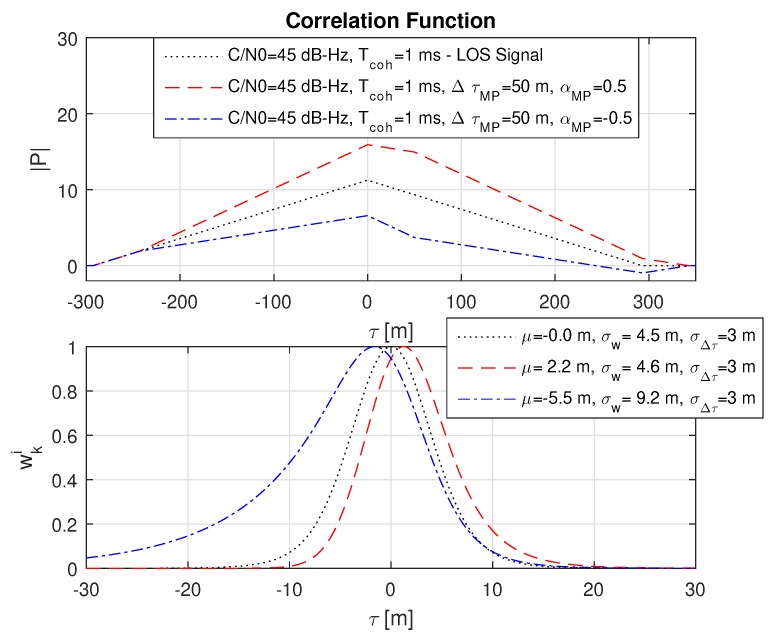
Influence of constructive and destructive multipath on the probability function with a relative multipath offset of $\Delta \tau_{MP}=50$ m and an amplitude of $\alpha_{MP}=0.5$ with respect to the line-of-sight (LOS) signal. The black dotted line refers to the LOS signal. It can be seen that the multipath variants shift the weighted mean μ in the opposite direction and that the destructive multipath significantly increases $\sigma_{w}$ due to the lower amplitude in |P|.

**Figure 10:**
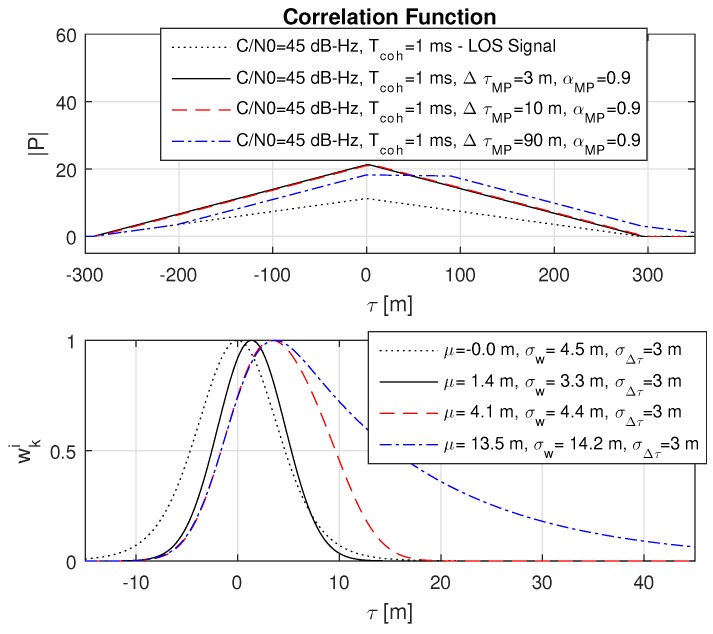
Impact of different multipath offsets $\Delta \tau_{MP}$ on the probability function. For a better visualization, a strong relative multipath to the LOS signal with $\alpha_{MP}=0.9$ is chosen. It can be seen that higher offsets increase the weighted mean μ but do not necessarily increase the weighted standard deviation $\sigma_{w}$. The dotted black line refers to the LOS signal.

The influence of constructive and destructive multipath is shown for a realistic case with a multipath amplitude of $\alpha_{MP}=0.5$ with respect to the LOS signal and a multipath offset of $\Delta \tau_{MP}=50$ m in Figure 9. As similar to the conventional Delay-Lock-Loop (DLL) in a GNSS receiver and thereof resulting pseudorange measurement, the weighted mean μ of the probability function also becomes biased. It can be seen that the resulting bias in μ is in the opposite direction for the constructive and destructive case. Furthermore, the destructive multipath reduces the amplitude of the correlation value |P|, which increases the weighted standard deviation $\sigma_{w}$, while the constructive multipath acts in the opposite direction and may also reduce the variance in distinct cases as visible in Figure 10 for the black line. Furthermore, it can be seen that constructive and destructive multipath changes the resulting probability function in a different way, even if the multipath parameters $\Delta \tau_{MP}$ and $\alpha_{MP}$ are exactly the same.

### 8.4. Impact of Short, Medium and Far Multipath

The impact on short, medium and far multipath on the probability function is shown on a strong multipath case $\alpha_{MP}=0.9$. Basically, it is expected that such a strong multipath seldomly occurs in typical real-world scenarios, but it was chosen to clearly visualize the impact of an increasing multipath offset. It can be seen in Figure 10 that an increase in $\Delta \tau_{MP}$ directly increases $\sigma_{w}$.

An interesting effect is the short constructive multipath case with an $\Delta \tau_{MP}=3$ m. In this case, the weighted standard deviation $\sigma_{w}$ is underestimated and shows a smaller variance than the LOS signal. This can be dangerous because this leads to a biased and at the same time more accurate measurement, compared to the truth. Considering the present multipath and incorporating it into the models for the weighting may prevent such an underestimation.

### 8.5. Impact of Different Multipath Amplitudes

**Figure 11:**
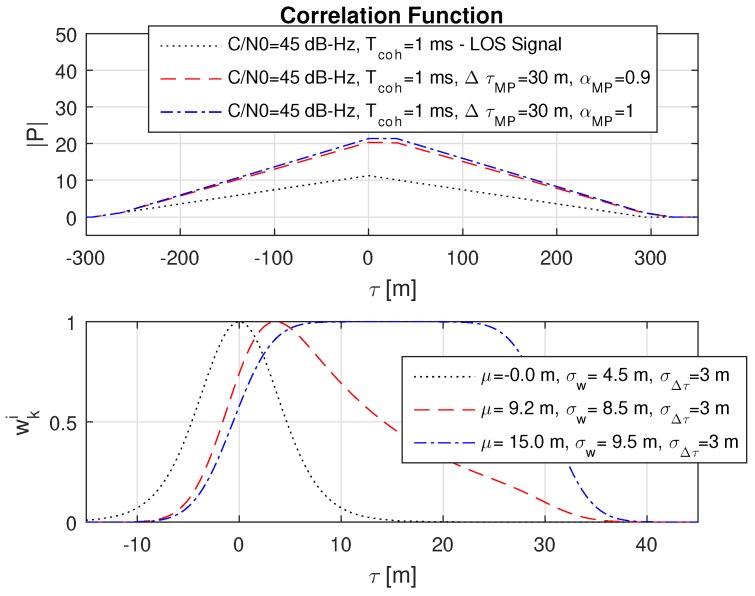
An increase of the multipath amplitude $\alpha_{MP}$ shifts the weighted mean μ and increases the weighted standard deviation $\sigma_{w}$. The probability function naturally covers the uncertainty also in the case of $\alpha_{MP}=1$, when the multipath signal is as strong as the LOS signal. The dotted black line refers to the LOS signal.

It is expected that an increase of the multipath amplitude $\alpha_{MP}$ further shifts the mean and increases the variance. This case is outlined in Figure 11. Naturally, in the case that the multipath signal is as strong as the LOS signal, the resulting probability function automatically covers the whole uncertainty range with a mean exactly between the two signals. This case is shown with the dash-dotted blue line in Figure 11.

### 8.6. Real-World Open Sky and Urban Scenario

The real-world tests evaluate the probability function with realistic data. Therefore, two scenarios have been selected: (1) an open sky case on a rooftop antenna in order to see the probability function under ideal conditions and (2) an urban case, where multipath is present and a significant shift and deformation on the probability function should be present.

The data was recorded in Graz, Austria and post-processed with the SX3 software receiver. The receiver was configured to dump the multi-correlator maps to files, which contain the correlation values |P|, which are related to the latest PVT. The multi-correlator maps contain correlation values for a range of code phases τ and Dopplers $f_{d}$, as shown in (**3**) of Figure 1. For all real-world tests, the red crosses in the upper plots correspond to the correlation values along the Doppler bin, which contains the correlation maxima. In order to obtain a high-resolution probability function, the correlation values (red crosses) have been interpolated with sinc function (black line).

**Figure 12:**
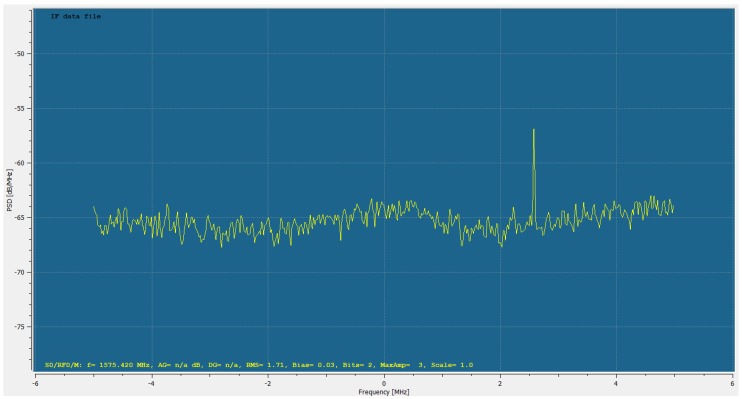
Frequency spectrum of the GPS L1 band with a root-mean-square (RMS) of the samples evaluating to $\beta_{s_{\mu },RMS}=1.71$. At frequency offset of approximately 2.5 MHz a continuous wave (CW) interference peak is present. The dataset was recorded with a measurement van during a measurement campaign. Based on the CW interference being present during the complete measurement run, it is assumed that it was caused by one of the active on-board measurement instruments or radio connections. Additionally, it is assumed that the present CW interference does not influence the measurement because it is significantly outside the main lobe of the analysed GPS L1 C/A (Coarse/Acquisition) signal.

It is of major importance that the input correlation values |P| are of correct amplitude because it basically drives the shape of the probability function and variance estimate. In order to obtain the same amplitude for |P| as in Equation (15), all amplitude scaling elements have been verified in the receiver. The GNSS Signal Samples ${\bar{s}}_{\mu }$ in Equation (15) have been recorded with a quantization of 2 bits in the Analog to Digital Conversion (ADC) stage. The two bits refer to the value range [−3 −1 1 3] and thus the RMS of the recorded samples differs from that in Equation (15) because the Automatic Gain Control (AGC) within the receiver front-end steers the amplitude of the GNSS signal in a way to optimally use the available quantization range. The RMS value of the samples ${\bar{s}}_{\mu }$ can be measured by the receiver. In this experiment, the RMS for the GPS L1 band was measured with $\beta_{s_{\mu },RMS}=1.71$, as also shown in Figure 12. Due to computational efficiency, the replica signal used for the correlation was generated with an amplitude of 8, thus it is simply $\beta_{rep}=8$. Based on this, the correlation values can be obtained after applying the scaling factors as

(52) $$\left|P\left(\tau ,\omega \right)|=\right|P_{MC,map}\left(\tau ,\omega \right)|\;{\left(\beta_{s_{\mu },RMS}\;\beta_{rep}\right)}^{-1},$$

where $|P_{MC,map}\left(\tau ,\omega \right)|$ refers to the correlation value from the multi-correlator map of the software receiver.

**Figure 13:**
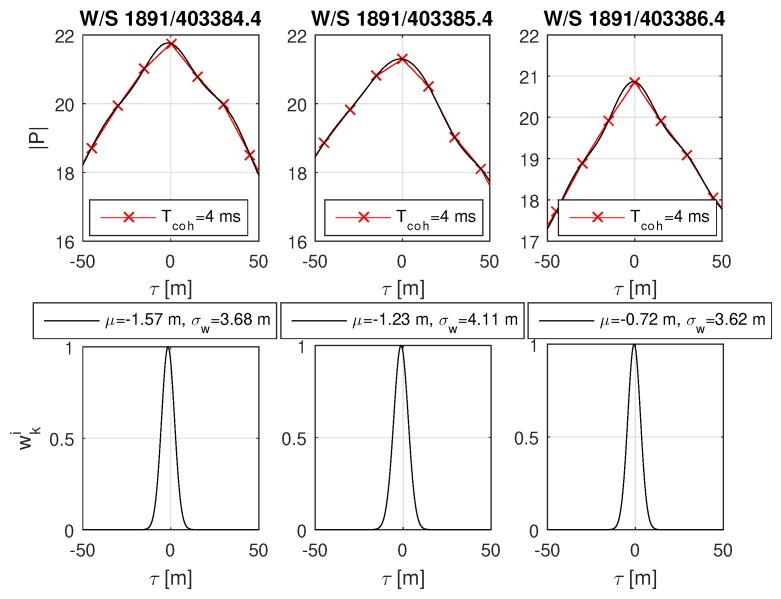
Real-world open sky scenario of satellite GPS L1 C/A Pseudo-Random-Noise (PRN) number 12. The plots show from left to right three epochs referring to GPS Week/Second W/S. The weighted standard deviations $\sigma_{w}$ approach the theoretical lower limit of $\sigma_{\Delta \tau }=3$ m for this ideal case. The dataset was recorded on the roof at LAT = 47.06446263 deg, LON = 15.40777110 deg at the Reininghausstraße 13a, Graz, Austria. The red crosses in the upper plots show the correlation values at code offset τ. The black line in the upper plot shows the sinc interpolated correlation values, which are used to obtain the weights $w_{k}^{i}$ shown in the lower plots.

For both scenarios, open sky and urban, the code delay bias standard deviation was set to $\sigma_{\Delta \tau }$ = 3 m and has been evaluated at three epochs. The open sky case is shown in Figure 13 and refers to good open sky conditions of GPS L1 C/A satellite PRN 12. The probability functions for all epochs show a slight bias of μ≈−1 m with respect to the feed back PVT. The weighted standard deviation $\sigma_{w}$ nearly approaches the theoretical lower limit of $\sigma_{\Delta \tau }=3$ m. No other significant effects are visible.

**Figure 14:**
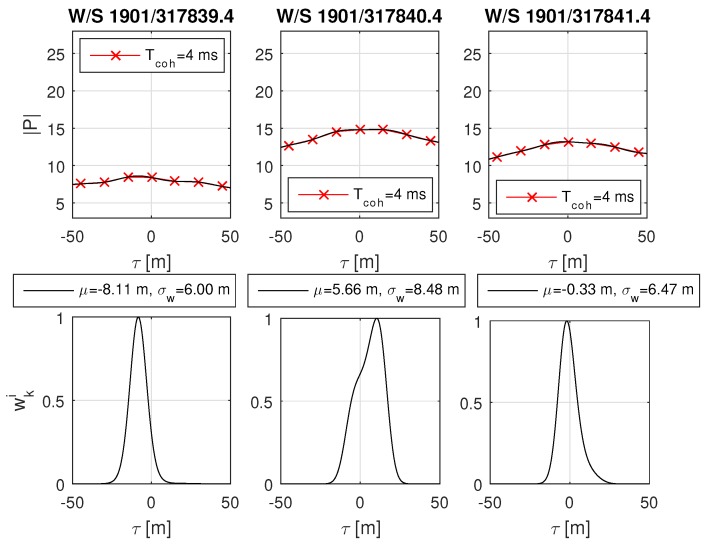
Real-world urban scenario of satellite GPS L1 C/A PRN 27. The plots show from left to right three epochs at different places in a urban environment, referring to the red dots from left to right in Figure 17. It is expected that the correlation values |P| vary significantly due to shadowing and multipath. The weighted mean is shifted for all positions and both the correlation and probability function of the second point (middle row) seem to be significantly affected by multipath. The red crosses in the upper plots show the correlation values at code offset τ. The black line in the upper plot shows the sinc interpolated correlation values, which are used to obtain the weights $w_{k}^{i}$ shown in the lower plots.

**Figure 15:**
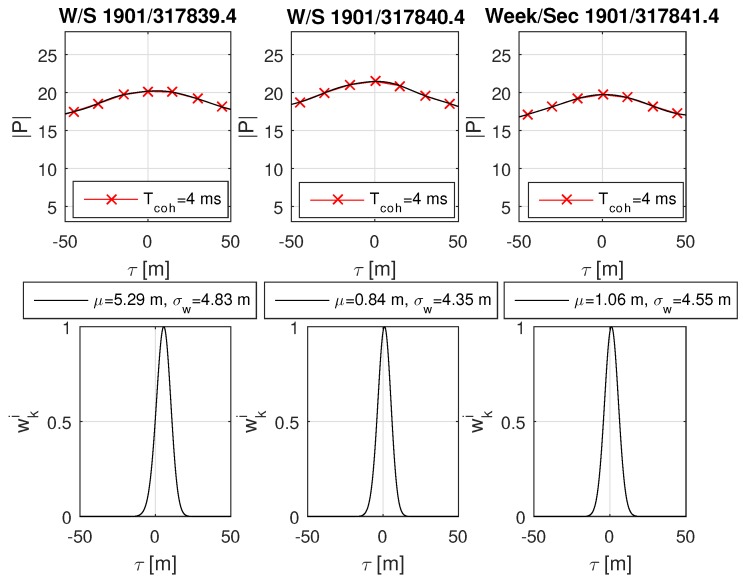
Real-world urban scenario of satellite GPS L1 C/A PRN 21. The plot content is analogical to Figure 14. For this satellite, it is assumed that the signal is less affected by the environment because there are fewer variations of the signal amplitude. Only in the case of the first position is the probability function biased.

**Figure 16:**
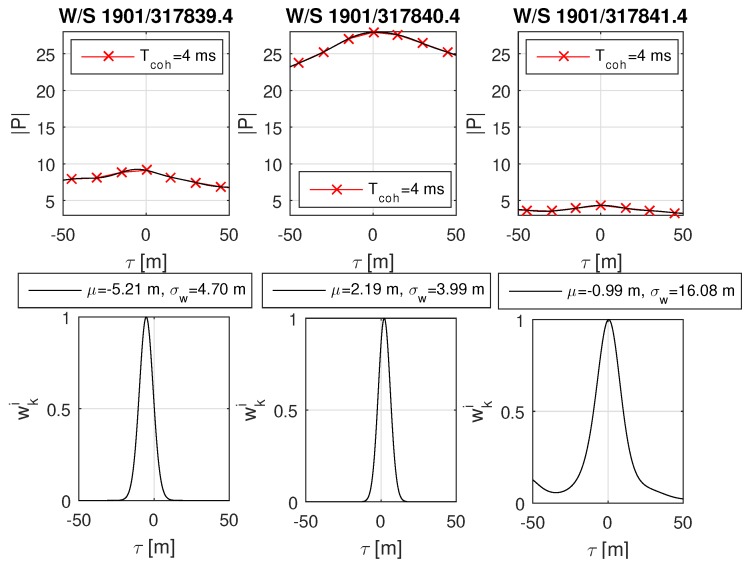
Real-world urban scenario of satellite GPS L1 C/A PRN 18. The plot content is analogical to Figure 14. This satellite signal is significantly affected by the environment. From the azimuth of satellite PRN 18 and the location of the buildings as shown in Figure 17, it can be assumed that the GNSS signal is blocked at the first and last positions, which fits to the amplitude of the correlation values. Interestingly, in the case of the third position, the significantly small correlation value leads to an increase in variance estimate.

**Figure 17:**
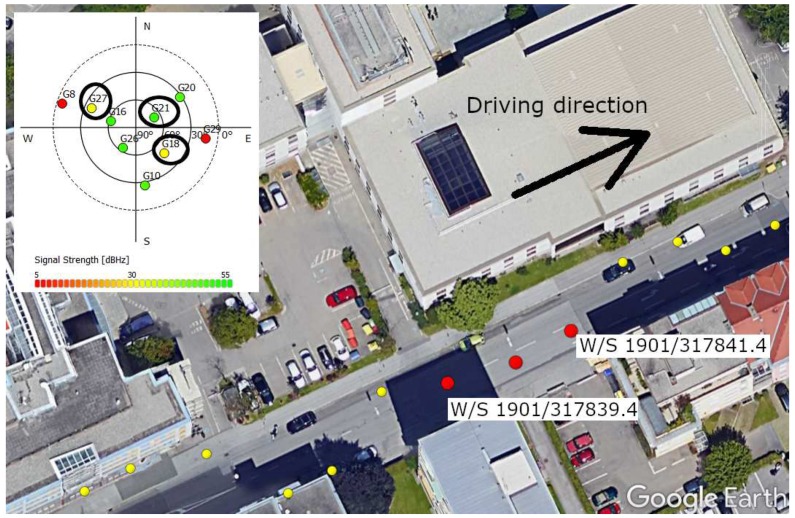
Environment for the urban scenario with three red measurement points. The driving direction was to the east, thus the red measurement points refer from left to the right column in Figure 14, Figure 15 and Figure 16. The upper left plot shows the satellite constellation. The three analyzed satellites PRN 27, PRN 21 and PRN 18 are marked with a black circle. The measurement was taken in the Steyrergasse in Graz, Austria. The point at W/S 1901/317839.4 refers to LAT = 47.06430622 deg, LON = 15.45391867 deg. Map image © 2017 Google, Landsat/Copernicus

The urban scenario was recorded during a test drive through Graz. The data has been post-processed and analyzed with the software receiver. An urban environment with significant deformation on the correlation function was selected and analyzed with MATLAB. The analysis focuses on the GPS L1 C/A satellites PRN 27, PRN 21 and PRN 18, as respectively shown in Figure 14, Figure 15 and Figure 16. A overview of the analyzed scenario is given in Figure 17, which shows the three analyzed positions (red dots) and the satellite constellation. Satellite PRN 27 in Figure 14 and satellite PRN 18 in Figure 15 show a significant variation in amplitude of the correlation values |P|, basically due to shadowing and multipath effects. Satellite PRN 21 in Figure 15 is less affected by the environment, because the signal amplitude remains nearly constant and no other significant effects are visible. Nearly all of the probability functions face a significant bias in μ and increased weighted standard deviation $\sigma_{w}$ compared to a standard open sky signal. Especially the weighted standard deviation of PRN 18 at third column in Figure 16 shows a significant increase due to the lowered correlation amplitude. Furthermore, the correlation function as well as the probability function of satellite PRN 27 in Figure 14, position at time W/S 1901/317840.4 (middle row) seem to be significantly affected by multipath, which is assumed due to the flattened peak in the correlation function and the resulting deformation of the probability function.

For clarification and as noted briefly above, all presented real-world scenarios show the resulting probability function with respect to the latest PVT estimate, not to an high accurate absolute reference. It should be considered that the latest PVT estimate might be already biased and thus the herein presented offset μ might not show the true bias. Dedicated analysis is planned for future studies using in one case a GNSS simulator and in another case a surveyed static reference position together with precise orbits. Nevertheless, the presented figures give an impression of the effects occurring (deformations and shifts) on the probability function. This is clearly visible when comparing the open sky case to the urban scenario, even if the biases can not be quantified with absolute values.

## 9. Conclusions

This paper discusses Bayesian Direct Position Estimation and describes a real-time capable implementation in a software based receiver. The Bayesian estimation framework is described in detail and a link to direct position estimation is established. The work shows the derivation of the optimal particle weight for BDPE and delivers a solution for performing the weight update in the logarithmic scale to overcome limited precision of digital computation. It further points out problems within Bayesian filters when performing the optimal particle weight update directly. Therefore, the paper proposes a method that introduces an additional nuisance parameter on the code measurement to cover the residual user range errors. The impact on several scenarios for a single GPS C/A BPSK(1) signal is given. These scenarios include a brief discussion on the impact of different signal strengths, different code delay bias variances, constructive and destructive multipath and different multipath amplitudes. Additionally, two real-world examples show the results of the optimal particle weighting applied on ideal open sky data and on multipath-prone urban environment data.

The work extends the original particle filter from [23], which followed a heuristic approach for the particle filter weight update. The original filter used a suboptimal chosen exponential weight update function, which causes a dense concentration of the particle cloud. In order to avoid such a dense concentration, the process noise was increased to cover also the residual user range errors. This is a working and viable approach to cover these errors, but it has caused rapid resampling steps of the particle filter.

From the proposed method, it is expected that a Bayesian filter can perform an optimal particle weight update with numerical stability. Furthermore, it is expected that the particle filter positioning performance and robustness will improve. The positioning benefits from the optimal particle weight update and it is expected that the estimated position becomes more accurate and the estimated accuracy of the position becomes more reliable. The introduced nuisance parameter allows for overlapping GNSS signals in the PVT domain in the case of unmodeled residual user range errors, which is expected to make, on one hand, the filter more robust and, on the other hand, reduce the number of resampling steps.

## Abbreviations

DPE	Direct Position Estimation
BDPE	Bayesian Direct Position Estimation
GNSS	Global Navigation Satellite System
PVT	Position, Velocity and Time
BPSK(1)	Binary Phase Shift Keying (with 1.023 MHz code rate)
SMC	Sequential Monte Carlo
EKF	Extended Kalman Filter
LTE	Long-Term Evolution
LOS	Line of Sight
VTL	Vector Tracking Loop
MC	Multi-Correlator
GPS	Global Positioning System
PF	Particle Filter
C/A	Coarse/Acquisition codes
RF	Radio Frequency
IF	Intermediate Frequency
ADC	Analogue to Digital Converter
USB	Universal Serial Bus
FFT	Fast Fourier Transform
CRLB	Cramér–Rao Lower Bound
AGC	Automatic Gain Control
CW	Continuous Wave
BW	Bandwidth
LAT	Latitude
LON	Longitude
RMS	Root Mean Square
PRN	Pseudo-Random-Noise
